# The New Application of UHPLC-DAD-TOF/MS in Identification of Inhibitors on β-Amyloid Fibrillation From *Scutellaria baicalensis*

**DOI:** 10.3389/fphar.2019.00194

**Published:** 2019-03-18

**Authors:** Lu Yu, An-Guo Wu, Vincent Kam-Wai Wong, Li-Qun Qu, Ni Zhang, Da-Lian Qin, Wu Zeng, Bin Tang, Hui-Miao Wang, Qiong Wang, Betty Yuen-Kwan Law

**Affiliations:** ^1^State Key Laboratory of Quality Research in Chinese Medicine, Macau University of Science and Technology, Taipa, Macau; ^2^Laboratory of Medical Chemistry, Department of Chemistry, School of Basic Medical Sciences, Southwest Medical University, Luzhou, China; ^3^Sino-Portugal Traditional Chinese Medicine International Cooperation Center, Southwest Medical University, Luzhou, China; ^4^Sichuan Key Laboratory of New Drug Discovery and Drugability Evaluation, Southwest Medical University, Luzhou, China; ^5^Luzhou Key Laboratory of Bioactivity Screening in Traditional Chinese Medicine and Drugability Evaluation, Southwest Medical University, Luzhou, China; ^6^Affiliated Traditional Chinese Medicine Hospital, Southwest Medical University, Luzhou, China; ^7^School of Pharmacy, Southwest Medical University, Luzhou, China

**Keywords:** Alzheimer’s disease, β-amyloid, fibrillation inhibitors, *Scutellaria baicalensis*, UHPLC-DAD-TOF/MS

## Abstract

Literary evidence depicts that aggregated β-amyloid (Aβ) leads to the pathogenesis of Alzheimer’s disease (AD). Although many traditional Chinese medicines (TCMs) are effective in treating neurodegenerative diseases, there is no effective way for identifying active compounds from their complicated chemical compositions. Instead of using a traditional herbal separation method with low efficiency, we herein apply UHPLC-DAD-TOF/MS for the accurate identification of the active compounds that inhibit the fibrillation of Aβ (1-42), via an evaluation of the peak area of individual chemical components in chromatogram, after incubation with an Aβ peptide. Using the neuroprotective herbal plant *Scutellaria baicalensis* (SB) as a study model, the inhibitory effect on Aβ by its individual compounds, were validated using the thioflavin-T (ThT) fluorescence assay, biolayer interferometry analysis, dot immunoblotting and native gel electrophoresis after UHPLC-DAD-TOF/MS analysis. The viability of cells after Aβ (1-42) incubation was further evaluated using both the tetrazolium dye (MTT) assay and flow cytometry analysis. Thirteen major chemical components in SB were identified by UHPLC-DAD-TOF/MS after incubation with Aβ (1–42). The peak areas of two components from SB, baicalein and baicalin, were significantly reduced after incubation with Aβ (1–42), compared to compounds alone, without incubation with Aβ (1–42). Consistently, both compounds inhibited the formation of Aβ (1–42) fibrils and increased the viability of cells after Aβ (1–42) incubation. Based on the hypothesis that active chemical components have to possess binding affinity to Aβ (1–42) to inhibit its fibrillation, a new application using UHPLC-DAD-TOF/MS for accurate identification of inhibitors from herbal plants on Aβ (1–42) fibrillation was developed.

## Introduction

Population aging is becoming a major demographical and health issue in the 21st century. Consistently, the number of people with aged-related neurodegenerative diseases, including Alzheimer’s disease (AD), Parkinson’s disease (PD), and Huntington’s disease (HD) are accelerating rapidly (Sheikh et al., 2013). The number of people with AD, the most common type of dementia (Fang et al., 2017), is expected to reach 74.7 and 131.5 million globally by 2030 and 2050, respectively (Alzheimer’s Disease International, 2018). The extracellular plaque of the Aβ peptide, and the neurofibrillary tangles (NFTs) composed of the microtubule associated filamentous tau protein in the brain, are two important pathological hallmarks of AD (Bloom, 2014). The accumulation of Aβ in neurons is recognized as the initiation step of AD progression, with the induction of oxidative stress, inflammation, and cell death (Di Bona et al., 2010). Although it remains controversial, Aβ is considered as one of the key targets for the treatment of AD (Yoshiike et al., 2003). Considering the fact that many natural small molecules have been proven to inhibit Aβ fibrillation and reduce the cytotoxicity of neurons, recent studies have focused on the screening of natural compounds with anti-AD effects in TCMs. For example, natural polyphenols such as curcumin can bind Aβ (1–42) and block its fibril formation and aggregation (Yang et al., 2005); resveratrol is capable of changing the oligomeric conformation and attenuate the cytotoxicity of Aβ (Feng et al., 2009); epigallocatechin-3-gallate is able to reduce the level of Aβ in brain and protect the mitochondrial function of neurons in the amyloid precursor protein (APP) and presenilin-1 (PS1) double transgenic AD mice (Dragicevic et al., 2011).

*Scutellaria baicalensis* (SB) (Huangqin) is a widely used TCM (Mehlhorn et al., 2014), which was firstly described in “Shen Nong Ben Cao Jing” (Yuan et al., 2015). Modern pharmacological studies have depicted its positive effects in neuroprotection (Yune et al., 2009; Miao et al., 2014), anti-cancer (Ye et al., 2002; Sato et al., 2013), anti-inflammation (Huang et al., 2006; Kim et al., 2009; Yoon et al., 2009), anti-oxidation (Gabrielska et al., 1997; Huang et al., 2006; Wang et al., 2014), anti-bacteria, and anti-virus (Zandi et al., 2013; Shi et al., 2016). Flavonoids including baicalin, baicalein, wogonin, oroxylin A-7-*O*-glucuronopyranoside, and oroxylin A are the major chemical components in SB (Lee et al., 2014). While baicalin, baicalein and wogonin were reported to inhibit fibrillation of Aβ (Heo et al., 2004; Zhu and Wang, 2015), total flavonoids from the stem and leaf of SB might improve learning or memory impairment and attenuate neuronal loss induced by the Aβ peptide in rats (Wang et al., 2013). However, the traditional bioactivity guided separation of active compounds from TCMs, requires repeated rounds of isolation and bioactivity validation before a single active fraction or chemical component can be identified. Due to the disadvantages of being time-consuming and laborious, the traditional way of identifying novel inhibitors on fibrillation of Aβ from TCMs is ineffective with slow progress (Gerardo Castillo et al., 2007; Liu et al., 2011; Kandasamy et al., 2012).

With the recent advances in the development of chemical analysis technologies, such as HPLC coupled with ultraviolet (UV) or florescence detector, mass spectrometry (MS) and nuclear magnetic resonance (NMR), identification of the bioactive component through the analysis of chromatograms, has become more prevalent (Li and Lurie, 2014; Wu et al., 2015; Bertini et al., 2017). Recent literature has reported a novel method on the structure-based discovery of fiber-binding compounds using computational docking, which was then validated by NMR for their binding affinity to both Aβ (16–21) and Aβ (1–42). The results confirmed that compounds with a binding affinity to Aβ, decreased toxicity of fiber by increasing its stability (Jiang et al., 2013). Consistently, a model using NMR for the validation of the binding affinity of non-natural peptides to tau fibers, showed a diminished^1^H NMR spectrum of the effective peptides (Sievers et al., 2011). Based on these observations, we hypothesized that chemical components would show a reduction in the UHPLC-DAD-TOF/MS chromatogram peak area upon effective Aβ binding. To validate the hypothesis, SB was selected as the study subject for the evaluation of method accuracy. Our results confirmed that 13 major chemical components were identified from the total ethanol extract of SB (SB-TEE). Among them, two of the chemical components, baicalin and baicalein showed a significant decrease of the peak area in the extracted-ion chromatogram (EIC) as evaluated by UHPLC-DAD-TOF/MS. Consistently, SB-TEE, baicalin and baicalein possess an inhibitory effect on the Aβ (1–42) fibril formation as validated by biolayer interferometry analysis, ThT fluorescence detection assay, native gel and dot blot analysis. The anti-fibrillation effect on Aβ was further confirmed by the decrease in its cytotoxicity after treatment with baicalin and baicalein. The current study presents a new approach using the UHPLC-DAD-TOF/MS system for the accurate screening or the quality control of anti-fibrillation compounds from TCMs, which may facilitate the drug discovery of potential anti-AD agents in the future.

## Materials and Methods

### Chemicals and Reagents

Baicalin, wogonoside and baicalein (≥98% purity, HPLC) were obtained from Chengdu MUST Bio-technology Company Ltd. (Chengdu, China). SB with the place of origin in Ji Lin province of China was purchased from Beijing Tong Ren Tang Zhuhai Pharmacy Co., Ltd. (Guangdong, China). 3-(4,5-dimethylthiazol-2-yl)-2,5-dimethyltetrazolium bromide (MTT) and ThT were purchased from Sigma (St. Louis, MO, United States). Milli-Q water was prepared by the Milli-Q integral water purification system (Millipore, Billerica, MA, United States) in our laboratory. Acetonitrile was purchased from Anaqua Chemicals Supply (Houston, TX, United States). Aβ (1–42) was obtained from the China Peptides Co., Ltd. (Shanghai, China). EZ-Link NHS-LC-LC-Biotin was obtained from Thermo Scientific Waltham (MA, United States). Super Streptavindin (SSA) biosensors were purchased from FortéBIO, PALL Life Sciences (Port Washington, NY, United States). The Annexin V staining kit was purchased from BD Biosciences (San Jose, CA, United States). The native PAGE Bis-Tris Gel (4–16%), running buffer (20X) and the native PAGE sample buffer were obtained from Invitrogen (Carlsbad, CA, United States). The PVDF membrane was obtained from PALL Life Sciences (Port Washington, NY, United States).

### Preparation of SB-TEE

25 gram of the SB plant was smashed into powder and extracted with 10 times its volume of 75% ethanol for 2 h, by refluxing two times. The extracted solution was then filtered, concentrated and dried with a rotary evaporator under reduced pressure to produce the final SB-TEE. The dried ethanol extracts were re-dissolved in DMSO at a suitable concentration for further use.

### Instrument and Chromatographic Conditions

UHPLC (Agilent Technologies 1290 Series), equipped with the time of flight (TOF) MS (Agilent Technologies 6230) with a jet stream ion source, was operated in negative and positive ion modes during the UHPLC analysis. All samples were analyzed using the Agilent Eclipse Plus C-18 column (100 × 2.1 mm) with a particle size of 1.8 μm at a flow rate of 0.35 mL/min. The separation was conducted in accordance with the gradient elution program, comprised of mobile phase A (0.1% formic acid in water) and mobile phase B (0.1% formic acid in ACN): 0–2 min, 2% B; 2–5 min, 2–10% B; 5–15 min, 10–50% B; 15–18 min, 50–80% B; 18–20 min, 80–100% B; 20–22 min, 100% B; 22.1–25 min, 2% B. For UHPLC-DAD-TOF-MS analysis, the data was acquired using the UV detector with the detection wavelength at 254 nm and in the scan mode with a m/z value from 100 to 1700 Da by 2.0 spectra/s. Data were analyzed using Agilent MassHunter Workstation software B.01.03.

### Aβ (1–42) Peptide Preparation

1 mg of Aβ peptide (1–42) was dissolved in 400 μL of hexafluoroisopropanol (HFIP; Sigma) and subjected to ultrasonic for 15 min. Aβ peptide solution was aliquoted into a 1.5 mL tube (100 μL/tube) and dried under a stream of nitrogen gas to produce a peptide film which was stored at -80°C. Aβ (1–42) was re-dissolved in 10 μL of DMSO (Sigma, United States) and an appropriate volume of PBS (pH = 7.4) to acquire the final concentration before use. The Aβ (1–42) peptide was then incubated at 37°C for 5 days to form the aggregated form of Aβ for all biological and chemical assays.

### Biolayer Interferometry Analysis

200 μL of solution containing 100 μg of the Aβ peptide was incubated at 37°C for 5 days. EZ-Link NHS-LC-LC-Biotin (Thermo Scientific, United States) was dissolved in DMSO to a concentration of 10 mM. Aβ (1–42) was biotinylated in a 1:0.5 molar ratio of biotin reagent and incubated for 30 min at room temperature before being added into a 96-well plate (Greiner Bio-One, PN:655209). Biotinylation was ascertained by loading the mixture onto super streptavidin (SSA) capacity tips (ForteìBIO, Menlo Park, CA, United States) and detected by the FortéBIO Octet Red instrument. Additionally, SSA biosensors were pre-wetted with PBS for the recording of baselines. Successful biotinylated Aβ (1–42) solution was collected and immobilized onto SSA tips overnight at 4°C. Compounds (baicalin and baicalein) dissolved in DMSO were diluted to an appropriate concentration with PBS to a final volume of 200 μL/well. Control wells were added with an equal amount of DMSO. All experiments consisted of repeated cycles of four major steps: wash (300 s), baseline (120 s), association (120 s), and dissociation (120 s). The results including the association and dissociation plot and kinetic constants were analyzed with ForteìBIO data analysis software.

### Thioflavin-T (ThT) Fluorescence Assay

20 μL of Aβ (1–42) (100 μM) was diluted with PBS or the tested SB compounds, to a final volume of 100 μL with 5 days of incubation at 37°C. A ThT fluorescence assay was performed as described in the previous report (Lu et al., 2011). Briefly, ThT was dissolved with PBS (pH = 7.4) at a final concentration of 20 μM and was kept away from light. 10 μL of aggregated Aβ with or without the tested compounds and 190 μL of ThT solutions were added into a black 96-well-plate and incubated for 1 h. Fluorescence measurements were carried out using the microtiter plate reader (SpectraMax Paradigm, Molecular Devices, United States) with excitation at 450 nm and emission at 490 nm. Background fluorescence was measured in the control sample containing PBS and 0.02% of DMSO.

### Dot Blot Assay

20 μM of Aβ (1–42) was incubated with SB-TEE or SB single compounds for 5 days at 37°C. 4 μL of each incubated solution was spotted onto the methanol pre-activated PVDF membrane, which was then blocked with 5% non-fat dried milk in Tris-buffered saline and Tween 20 (TBST) for 1 h. The membrane was incubated with primary anti-amyloid fibril antibody [mOC87] (1:1000) (Abcam, Cambridge, MA, United States) overnight at 4°C, followed by an incubation with HRP-conjugated secondary antibody. Protein bands were detected using ultra signal sensitive ECL Western Blotting detection reagent (4A Biotech Co., Ltd, Beijing, China) and visualized using gel imaging equipment (Amersham Imager 600, GE, Tokyo, Japan). Band intensity was quantified using the software ImageJ (National Institutes of Health, Bethesda, MD, United States).

### Native Gel Electrophoresis

After the incubation of Aβ (1–42) with SB-TEE or SB single compounds at 37°C for 5 days, with Aβ (1–42) alone set as the control group, the incubated solutions were centrifuged at 10,000 *g* for 10 min. The supernatant was collected for the detection of soluble Aβ (1–42) oligomers by native gel electrophoresis. In brief, samples in the Novex native PAGE sample buffer were loaded into the pre-casted native PAGE gels for electrophoresis in 1X of Novex Native PAGE running buffer. The proteins on the gel were then transferred to a PVDF membrane. The membrane was blocked with 5% non-fat milk in TBST and then immunoblotted with an antibody against amyloid fibril [mOC87] (1:1000) (Abcam, Cambridge, MA, United States) overnight at 4°C. After an incubation with HRP-conjugated secondary antibody, protein bands were detected and visualized as described in the previous “Dot Blot Assay” section.

### Cell Viability

PC-12 cells were cultured with DMEM (Gibco, Grand Island, NY, United States) containing 10% horse serum (Gibco, Grand Island, NY, United States), 5% fetal bovine serum (FBS, PAN Biotech, Germany) and 1% penicillin and streptomycin, in a humidified incubator with 5% CO_2_ at 37°C. Cell viability of PC-12 cells was measured using MTT method (Wong et al., 2005). In brief, PC-12 cells plated on 96-well plates were incubated with Aβ (1–42) alone, Aβ (1–42) with SB-TEE or Aβ (1–42) with single compounds from SB, respectively. After 48 h of treatment, 10 μL of MTT solution (Sigma, United States) was added to cells in each well and further incubated for 4 h at 37°C. The incubation medium was then removed and 150 μL of DMSO was added to cells to dissolve the formazan. Absorbance (OD) of each well was then detected by spectrophotometer at the wavelength of 490 nm. The percentage of cell viability was calculated using the formula: cell viability (%) = cells number_(treated)_/cells number _(DMSOcontrol)_ × 100 %. Data were obtained from 3 independent experiments.

### Flow Cytometry Analysis

Cell viability of PC-12 cells was further evaluated by flow cytometry using the annexin V staining kit (BD Biosciences, San Jose, CA, United States). In brief, PC-12 cells seeded in a 6-well-plate were treated with Aβ (1–42) with or without the addition of SB-TEE or its single compounds, for 48 h. After treatments, the cells were trypsinized and centrifuged. The cells pellets were re-suspended with 250 μL of PBS and then stained with 2 μL of propidium iodide and 1 μL of FITC (BD Biosciences, San Jose, CA, United States) for 15 min. The cells were then analyzed using a FACSCalibur flow cytometer (BD Biosciences, San Jose, CA, United States). Data acquisition and analysis were performed using the Flowjo 7.6.1 (TreeStar, San Carlos, CA, United States).

### Statistical Analysis

All analyses were performed using the GraphPad Prism 6.0 (GraphPad Software, Inc., San Diego, CA, United States). All data were presented as means ± SEM. The difference was considered to have statistical significance if *p* < 0.05. Student’s *t*-test or one-way ANOVA was applied for statistical analysis to compare all the different groups in the current study.

## Results

### Characterization of the Chemical Components in the Total Ethanol Extract of *Scutellaria baicalensis*

**FIGURE 1:**
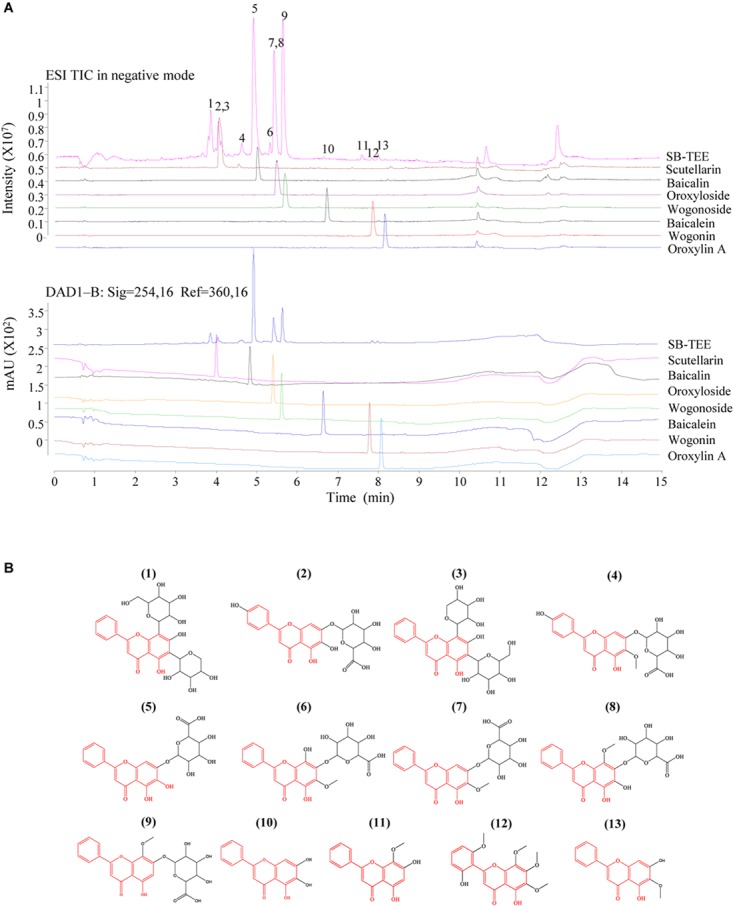
The identification of the chemical components in SB using Agilent 6230 UHPLC-DAD-TOF/MS. **(A)** TIC (total ion chromatogram) and the corresponding UV chromatogram of SB-TEE and its main components (scutellarin, baicalin, oroxyloside, wogonoside, baicalein, wogonin and oroxylin A). **(B)** The chemical structures of the identified components in SB.

SB is a rich source of polyphenols, especially flavonoids, which are a major class of bioactive components in SB (Zhou et al., 2015). Up to now, more than 100 polyphenols were identified from the genus *Scutellaria* (Sripathi and Ravi, 2017). Recent studies have reported the neuroprotective effects of SB and its flavonoids in anti-Aβ fibrillation and improvement of cognitive function (Heo et al., 2004; Yune et al., 2009; Wang et al., 2013). With its well-known neuroprotective effects, SB was selected as the subject of our study. To begin, the chemical components of SB were characterized using UHPLC-DAD-TOF/MS in negative or positive ion mode and UV at 254 nm. As shown in the total ion chromatogram (TIC) and UV chromatogram of SB-TEE in Figure 1A, a total of 13 peaks were observed and marked. Among them, the identities of 7 flavonoids including scutellarin (#2), baicalin (#5), oroxyloside (#7), wogonoside (#9), baicalein (#10), wogonin, (#11) and oroxylin A (#13) from SB-TEE, were confirmed with reference to the chromatograms of their corresponding standard pure compounds analyzed under the same chromatographic conditions. The identities of the remaining six components (#1, #3, #4, #6, #8, and #12) were confirmed by comparing their accurate mass and retention features on the C_18_ column, to the reported literature. With their common nuclear structure labeled in red, 13 major peaks were identified, with their chemical structures shown in Figure 1B. The retention time, formula, chemical name and the accurate MS in negative mode or positive mode are listed in Table 1. All the characterizations of the major chemical components in SB were confirmed before being further subjected to biological evaluation.

**Table 1:** The retention time, formula, chemical name and the mass of the chemical components in negative and positive ion modes.

Peak No.	Retention time (min)	Formula	Chemical name	[M-H]^-^ (m/z)		ppm	[M+H]^+^ (m/z)		ppm
							
				Calculated	Measured		Calculated	Measured	
1	3.91	C26H28O13	Chrysin -6-*C*-arabinose-8-*C*-glucose	547.1457	547.1474	3.11	549.1603	549.1641	6.92
2	4.081	C21H18O12	Scutellarin	461.0725	461.033	1.74	463.0871	463.0911	8.64
3	4.223	C26H28O13	Chrysin -6-*C*-glucose-8-*C*-arabinose	547.1457	547.1485	5.12	549.1603	549.1647	8.01
4	4.78	C22H20O12	Hispidulin 7-glucuronide	475.0882	475.0899	3.58	477.1028	477.105	4.6
5	5.046	C21H18O11	Baicalin	445.0776	445.0809	7.41	447.0922	447.0959	8.24
6	5.62	C22H20O12	5,7,8-Tetrahydroxyflavone; 6-Me ether, 7-*O*-β-D-glucuronopyranoside	475.0882	475.0917	7.37	477.1028	477.1063	7.34
7	5.562	C22H20O11	Oroxyloside	459.0933	459.097	8.06	461.1078	461.1116	8.24
8	5.656	C22H20O12	5,6,7-Tetrahydroxyflavone; 8-Me ether, 7-*O*-β-D-glucuronopyranoside	475.0882	475.0862	-4.2	477.1028	477.1035	1.46
9	5.762	C22H20O11	Wogonoside	459.0933	459.0975	9.15	461.1078	461.1109	6.72
10	6.748	C15H10O5	Baicalein	269.0455	269.0469	5.2	271.0601	271.0622	7.75
11	8.024	C16H12O5	Wogonin	283.0612	283.0614	0.71	285.0757	285.078	8.07
12	8.149	C19H18O8	Skullcapflavone II	373.0929	373.0929	0	375.1074	375.1103	7.73
13	8.291	C16H12O5	Oroxylin A	283.0612	283.0629	6	285.0757	285.0776	6.66


### Prediction of Aβ (1–42) Peptide-Binding Propensity of Selected SB Chemical Components by UHPLC-DAD-TOF-MS

**FIGURE 2:**
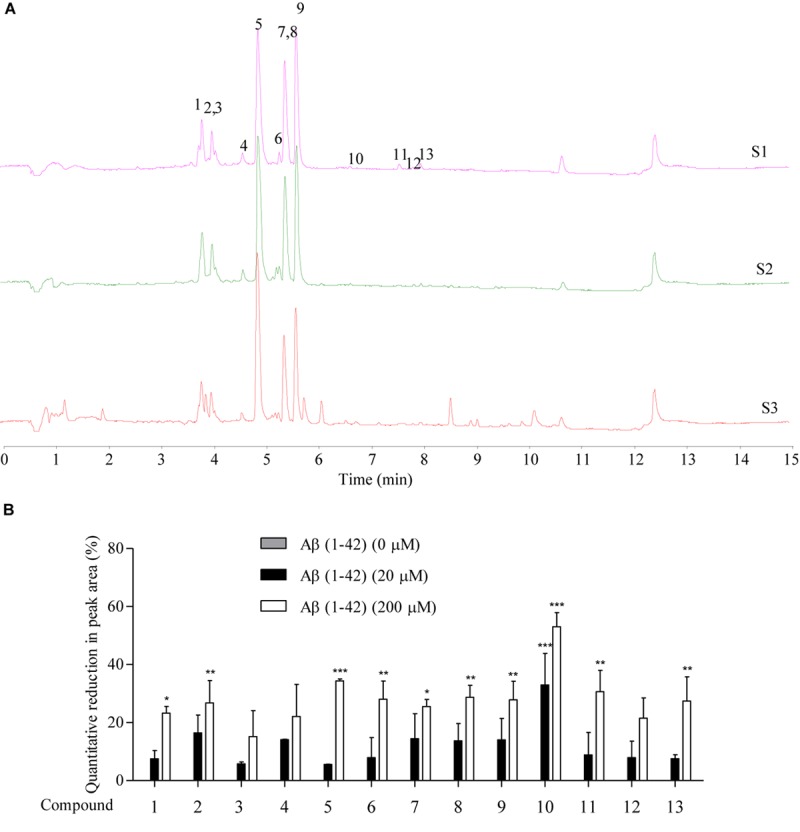
The prediction of Aβ-binding propensity of chemical components in SB using Agilent 6230 UHPLC-DAD-TOF/MS**. (A)** The TIC of SB-TEE with or without the incubation with 20 and 200 μM Aβ (1–42). S1: SB-TEE; S2: The incubation of SB-TEE with 20 μM Aβ (1–42); S3: The incubation of SB-TEE with 200 μM Aβ (1–42). **(B)** The bar chart shows the percentage of reduction (%) in the peak area of each SB component in TIC after their incubation with 20 or 200 μM Aβ (1–42). Columns means of 3 independent experiments; bars, SEM. ^∗^*p* < 0.05, ^∗∗^*p* < 0.01 and ^∗∗∗^*p* < 0.001 vs. 0 μM Aβ (1–42) group.

The Aβ peptide, which constitutes the amyloid plaques in the brains of AD patients, has long been identified as the major cause of AD. Upon sequential cleavage by the γ-secretase and β-secretase, Aβ can be produced from the APP with the major cleavage sites of γ-secretase at the positions 40 and 42 of the Aβ peptide. With its soluble toxic nature and high propensity to fibrillate and form amyloid plaques (Yiannopoulou and Papageorgiou, 2013; Xiao et al., 2015; Walti et al., 2016), accumulation of Aβ (1–42) is highly correlated with the pathogenesis of AD, with a cascade of cellular responses such as inflammation, oxidative damage and neurotoxicity (Graham et al., 2017). Therefore, therapeutic approaches that target the accumulated aggregation and fibrillation of Aβ with antibodies, peptides, or chemical molecules has intensively been investigated (Cho and Kim, 2011). To predict the peptide-binding propensity of the above 13 identified chemical components in SB, SB-TEE was incubated with 20 and 200 μM of Aβ (1–42), respectively. With a blank solution and SB-TEE without Aβ (1–42) incubation working as the control, all reaction product mixture was analyzed using UHPLC-DAD-TOF/MS under the same chromatographic conditions. Figure 2A shows the TIC of SB alone (S1), and SB-TEE incubated with 20 μM (S2) or 200 μM (S3) of Aβ (1–42), respectively. With the peak area of all the identified components calculated and analyzed, Figure 2B and Table 2 show the percentage (%) of decrease in the peak area of all the identified chemical components in TIC. Among them, baicalin (peak #5) and baicalein (peak #10) showed the highest reduction in peak area (34.38 and 53.03%) after incubation with Aβ (1–42) (200 μM).

**Table 2:** Quantitative reduction (%) of peak area of each SB component in TIC after their incubation with 20 or 200 μM Aβ (1–42).

Compounds in SB	Quantitative reduction of peak area (%)	
	
	With 20 μM Aβ (1–42)	With 200 μM Aβ (1–42)
1	7.53 ± 4.05	23.24 ± 3.33^∗^
2	16.41 ± 8.71	26.78 ± 10.92^∗∗^
3	5.79 ± 0.94	15.2 ± 12.59
4	14.11 ± 0.15	22.1 ± 15.63
5	5.58 ± 0.06	34.38 ± 0.96^∗∗∗^
6	7.87 ± 9.84	28.04 ± 8.81^∗∗^
7	14.40 ± 12.19	25.51 ± 3.51^∗^
8	13.72 ± 8.38	28.69 ± 5.85^∗∗^
9	14.03 ± 10.43	27.8 ± 9.05^∗∗^
10	32.94 ± 15.38^∗∗∗^	53.03 ± 6.93^∗∗∗^
11	8.81 ± 10.94	30.61 ± 10.39^∗∗^
12	7.89 ± 8.06	21.47 ± 9.9
13	7.59 ± 1.89	27.4 ± 11.81^∗∗^


*Note: ^∗^*p* < 0.05, ^∗∗^*p* < 0.01, and ^∗∗∗^*p* < 0.001 vs. 0 μM Aβ (1–42) group.*

### Validation of Aβ (1–42) Peptide-Binding Propensity of Selected SB Chemical Components by ThT Assay

**FIGURE 3:**
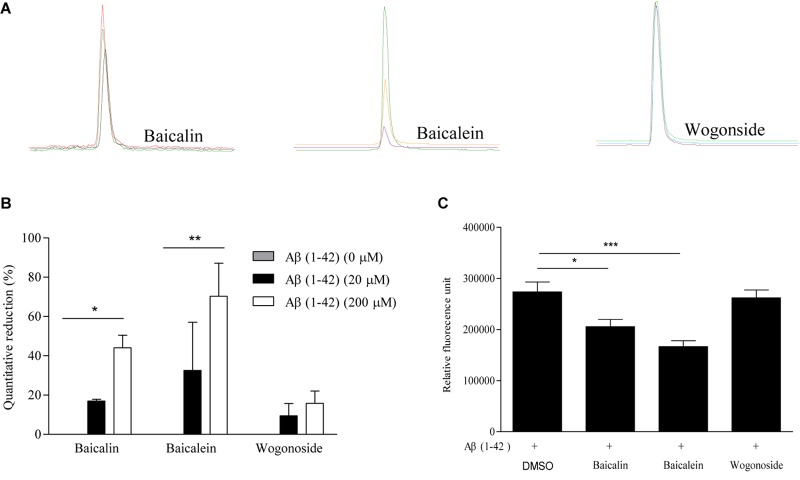
The validation of the Aβ-binding propensity of chemical components by ThT fluorescence assay. **(A)** The extracted-ion chromatogram (EIC) of the selected SB components (baicalin, baicalein, and wogonoside) after incubation with 20 and 200 μM Aβ (1–42). **(B)** The bar chart shows the quantitative reduction (%) in the peak area of baicalin, baicalein, and wogonoside in TIC after incubation with 20 and 200 μM Aβ (1–42). The data was expressed as the mean ± SEM. ^∗^*p* < 0.05 and ^∗∗^*p* < 0.01 vs. 0 μM Aβ (1–42). **(C)** The inhibitory effect of representative compounds on Aβ (1–42) fiber formation measured by ThT fluorescence intensity detection. The data was expressed as the mean ± SEM. ^∗^*p* < 0.05 and ^∗∗∗^*p* < 0.001 vs. 20 μM Aβ (1–42) alone group.

To further validate the peptide-binding propensity of selected SB compounds, baicalin (peak #5) and baicalein (peak #10), which showed the highest percentage of peak area reduction in TIC, were subjected to further validation with the negative control, wogonoside (peak #9), which showed no significant change in the peak area (Table 3). As shown in Figure 3A,B, the peak areas of baicalin and baicalein in TIC were negatively correlated to the concentrations of Aβ. Upon binding to amyloid fibrils, benzothiazole fluorescent dye (ThT) can produce a strong fluorescence signal at the wavelength of 482 nm (Xue et al., 2017), therefore, the widely used ThT is applied to further monitor and confirm the inhibition of Aβ fibril formation *in vitro*. As shown in Figure 3C, fluorescence intensity indicated that the concentration of Aβ fibril was decreased upon treatment with baicalin and baicalein, suggesting the positive role of baicalin and baicalein in anti- Aβ fibril formation.

**Table 3:** The percentage (%) of reduction in the peak areas of baicalin, baicalein and wogonoside in TIC after incubation with 20 or 200 μM Aβ (1–42).

Compound	Quantitative reduction of peak area (%)	
	
	With 20 μM Aβ (1–42)	With 200 μM Aβ (1–42)
Baicalin (50 μM)	16.96 ± 1.28	44.07 ± 9.03^∗^
Baicalein (50 μM)	32.52 ± 34.67	70.28 ± 23.84^∗∗^
Wogonoside (50 μM)	9.49 ± 8.81	15.77 ± 8.84


*Note: ^∗^*p* < 0.05 and ^∗∗^*p* < 0.01 vs. 0 μM Aβ (1–42) group.*

### Direct Binding Measurement of Baicalin and Baicalein to Aβ (1–42) by Biolayer Interferometry Analysis

**FIGURE 4:**
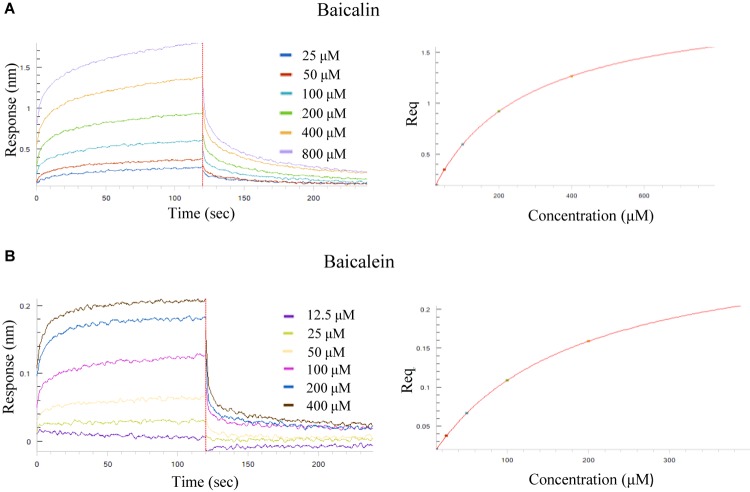
Kinetic measurement of the binding affinity of baicalin and baicalein to Aβ (1–42) fibril by BLI assay. **(A)** Kinetic binding sensorgrams of increasing concentrations of baicalin from 25 to 800 μM were showed with real-time data acquisition for each step of the kinetic assay. **(B)** Kinetic binding sensorgrams of increasing concentrations of baicalein from 12.5 to 400 μM. Response/binding (nm) represents the optical thickness of the sensor layer reflected by the spectral shift (Δλ) upon the interaction of baicalin or baicalein with Aβ (1–42). The response at steady state where the rate of association equal to the dissociation, the equilibrium binding signal (Req) indicated by flattened curve is reached.

To determine the binding affinity of the selected SB chemical components to Aβ (1–42) fibril, the label-free biolayer interferometry assay which measures the biomolecular interaction was used. It is performed by monitoring the binding of a ligand immobilized on the tip surface of a biosensor to a specific analyte in the tested solution. Upon the association, the optical thickness at the biosensor tip will be increased, leading to a real time shift in wavelength (Δλ) due to a change in the thickness of the biological layer. These Interactions will then be quantitated to provide accurate data on binding specificity and the association or dissociation rates. To begin, increasing concentrations of baicalin (25, 50, 100, 200, 400, 800 μM) and baicalein (12.5, 25, 50, 100, 200, 400 μM) were applied for the real-time monitoring of their direct association with the biotinylated Aβ (1–42). As shown by the association/dissociation binding curves of the 2 compounds in Figure 4A,B, there is a dose dependent increase in the optical thickness (nm) of the sensor layer, suggesting the direct binding of baicalin and baicalein to Aβ. Table 4 showed the kinetic constants calculated using the ForteìBIO data analysis software. The results showed that the *K*_D_ value of baicalin and baicalein were 242 and 170 μM, respectively, suggesting the direct and reversible interaction of baicalin and baicalein with Aβ (1–42).

**Table 4:** The binding affinity (KD), association rate constant (K_on_) and dissociation rate constant (K_dis_) of baicalin and baicalein to Aβ (1–42).

	KD (μM)	Kon (1/Ms)	Kdis (1/s)
Baicalin	242	1.66 × 10^+02^	4.01 × 10^-02^
Baicalein	170	9.22 × 10^+02^	1.57 × 10^-01^


### *In vitro* Inhibitory Effect of SB-TEE, Baicalin, Baicalein and Wogonoside on Aβ (1–42) Fibrillation

**FIGURE 5:**
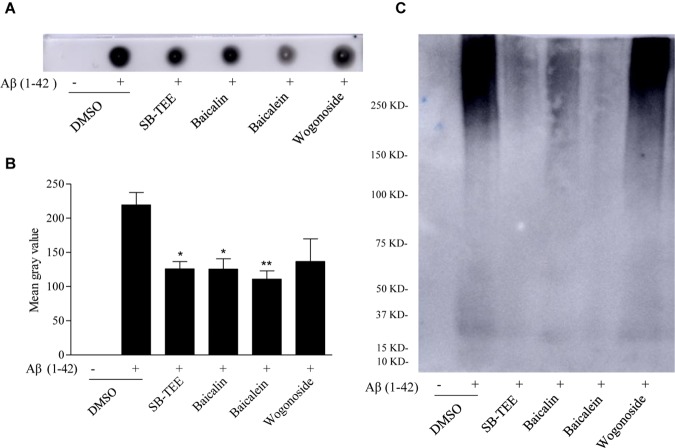
Inhibitory effect of SB-TEE and selected compounds on Aβ (1–42) fibrillation by dot blot assay and native gel electrophoresis analysis. **(A)** The dot blot image of Aβ (1–42) with or without the incubation with SB-TEE (100 μg/mL), baicalin (50 μM), baicalein (50 μM), or wogonoside (50 μM). **(B)** The dot blot images were analyzed and quantitated with the software Image J. Columns, means of 3 independent experiments; bars, SEM. ^∗^*p* < 0.05, ^∗∗^*p* < 0.01 vs. 20 μM Aβ (1–42) alone group. **(C)** The native gel electrophoresis analysis was performed by analyzing Aβ (1–42) solution (50 μg) with or without incubation of SB-TEE, baicalin, baicalein or wogonoside at 37°C for 5 days using the 4–16% gradient native gel under non-denatured condition. The full-length images of dot blot and native gel electrophoresis are displayed in Supplementary Figure S1.

Dot immunoblot, utilizing the strong Aβ-binding capacity of PVDF membrane, was used for further validation of the *in vitro* anti-Aβ (1–42) fibrillation effect of SB-TEE, baicalin, baicalein and wogonoside. To begin, incubation of 20 μM Aβ (1–42) with or without SB-TEE, baicalin, baicalein or wogonoside for 5 days at 37°C was performed. Equal amount of each incubated sample spotted onto the PVDF membrane was then subjected to immunoblotting with antibodies specific for detection and quantitation of amyloid fibrillation (Gong et al., 2003). As shown in Figure 5A,B, while baicalein possesses the highest potency in anti-fibrillation of Aβ, wogonoside was not effective in inhibiting the fibrillation of Aβ. To further confirm the anti-fibrillation effect of the selected compounds, non-denaturing native gel electrophoresis was applied for the effective detection of fibrillary Aβ. Consistently, baicalein showed the highest potency in anti-fibrillation of Aβ, as shown in Figure 5C. These data have confirmed the feasibility of our proposed new application of using a UHPLC-DAD-TOF/MS based detection system for precise identification of β-amyloid fibrillation inhibitors from TCMs that contain multiple chemical components.

### SB-TEE Alleviates the Cytotoxicity of Aβ (1–42) in PC-12 Cells

**FIGURE 6:**
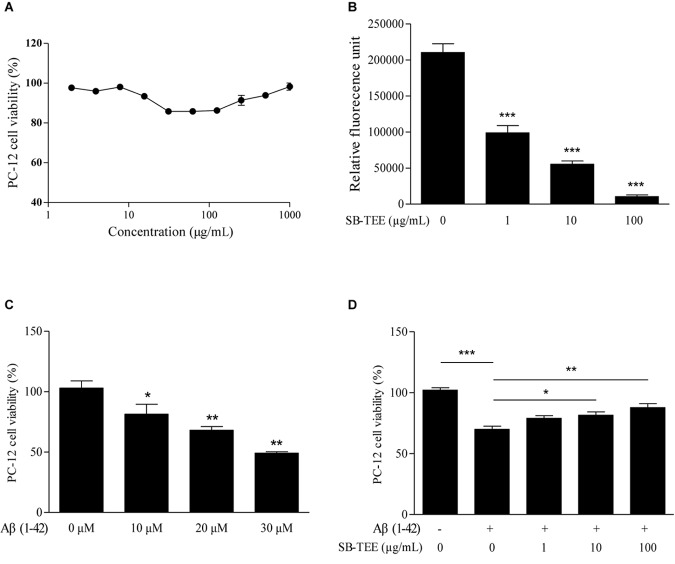
Cytotoxicity of SB-TEE and Aβ (1–42) fibril on PC-12 cells. **(A)** Cytotoxicity of SB-TEE on PC-12 was examined by MTT assay at 48 h after treatment. **(B)** The anti- Aβ fibrillation effect of SB-TEE (1–100 μg/mL) was measured by ThT fluorescence assay. ^∗∗∗^*p* < 0.001 vs. 20 μM Aβ (1–42) group. **(C)** Cellular toxicity induced by 10–30 μM of Aβ (1–42) on PC-12 cells was evaluated by MTT assay at 48 h after treatment. ^∗^*p* < 0.05 and ^∗∗^*p* < 0.01 vs. 0 μM Aβ (1–42) group. **(D)** Cellular toxicity induced by 20 μM of Aβ (1-42) on PC-12 cells was evaluated by MTT assay at 48 h after SB-TEE (1, 10, and 100 μg/mL) treatments. Column, means of 3 independent experiments; bars, SEM. ^∗^*p* < 0.05, ^∗∗^*p* < 0.01 and ^∗∗∗^*p* < 0.001 vs. 20 μM Aβ (1–42) alone group.

To further validate the correlation between the anti-fibrillation effect and the functional role of SB, the effect of SB-TEE on Aβ (1–42)-induced cell death was evaluated. To begin, the viability of PC-12 cells upon SB-TEE treatment, from 0 to 1000 μg/mL, was evaluated by MTT assay. As shown in Figure 6A, no obvious cellular toxicity was observed within the tested concentration range. Furthermore, fibrillation of Aβ (1–42) was performed in the presence of PBS or SB-TEE with 5 days of incubation at 37°C. A ThT fluorescence assay confirmed that the fibrillation of Aβ decreased with an increasing concentration of SB-TEE from 1 and 100 μg/mL, as shown in Figure 6B. To further elucidate the anti-Aβ fibrillation effect of SB-TEE in cells, viability of PC-12 cells incubated with an increasing concentration of Aβ (1–42), from 10 to 30 μM, was evaluated by MTT assay as shown in Figure 6C. With an incubation concentration of 20 μM Aβ (1–42), Figure 6D confirmed that an increasing concentration of SB-TEE decreased the toxicity of Aβ (1–42), which is correlated with the anti-Aβ fibrillation effect of SB-TEE. Our results have confirmed the protective role of SB-TEE on Aβ-induced toxicity in cells, which further validated the accuracy and precision of applying the UHPLC-DAD-TOF/MS detection system for the identification of Aβ fibrillation inhibitors, from analytical chemistry to cellular functional level.

### Protective Role of Baicalin, Baicalein and Wogonoside in the Cytotoxicity of Aβ (1–42) in PC-12 Cells

**FIGURE 7:**
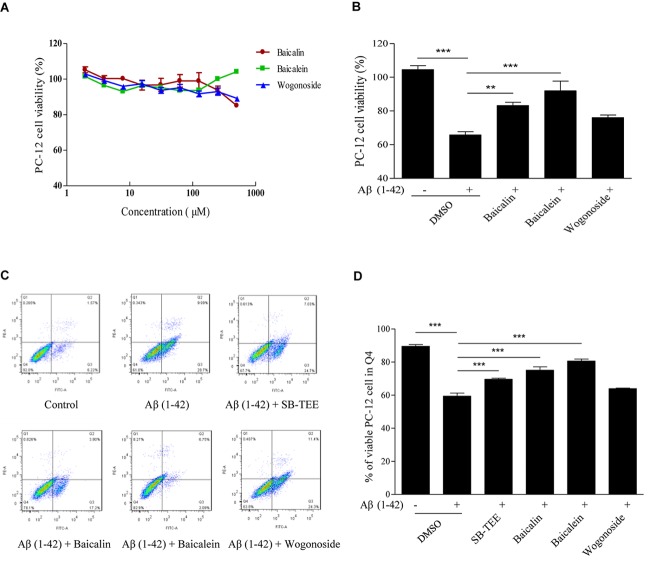
Aβ (1–42) fibril-induced cytotoxicity on PC-12 cells after baicalin, baicalein and wogonoside treatments. **(A)** Cytotoxicity of baicalin, baicalein and wogonoside (0–500 μM) on PC-12 were examined by MTT assay at 48 h after treatment. **(B)** Cellular toxicity induced by 20 μM of Aβ (1–42) on PC-12 cells was evaluated by MTT assay at 48 h after baicalin (50 μM), baicalein (50 μM), and wogonoside (50 μM) treatments. Columns means of 3 independent experiments; bars, SEM. ^∗∗^*p* < 0.01 and ^∗∗∗^*p* < 0.001 vs. 20 μM Aβ (1–42) group. **(C)** Cellular toxicity induced by 20 μM of Aβ (1–42) with or without the co-treatment of baicalin, baicalein and wogonoside on PC-12 cells was evaluated by flow cytometry at 48 h after treatment. **(D)** Quantitative results on the percentage of viable cells after SB-TEE, baicalin and baicalein treatments with the presence of Aβ (1–42) in PC-12 cells. Columns, means of 3 independent experiments; bars, SEM. ^∗∗∗^*p* < 0.001 vs. 20 μM Aβ (1–42) alone group.

Aβ (1–42)-induced oxidative stress and neurotoxicity have been implicated in the pathogenesis of AD (Butterfield, 2002). With the protective effect of SB-TEE in alleviating the cytotoxicity of Aβ (1–42) in PC-12 cells, the role of the selected potential anti-Aβ fibrillation herbal compounds, baicalin and baicalein, identified by UHPLC-DAD-TOF/MS were further evaluated by MTT and flow cytometry analysis. As shown by the MTT results in Figure 7A, baicalin, baicalein and wogonoside possess very low or no cytotoxicity in PC-12 cells at concentrations ranging from 0 to 100 μM. Viability of PC-12 cells incubated with Aβ (1–42) (20 μM) with or without the presence of baicalin (50 μM), baicalein (50 μM) or wogonoside (50 μM) were evaluated by MTT assay. As shown in Figure 7B, while both baicalin and baicalein were able to rescue cells from Aβ (1–42)-induced cell death, wogonoside showed no effect in the restoration of cell viability. The MTT result was further confirmed by flow cytometry analysis using annexin V staining. As shown by the increased percentage of viable cells in Figure 7C,D, quantitated flow cytometry results confirmed the protective role of baicalin and baicalein in Aβ (1–42)-induced cell death. All these data have further supported the notion of applying UHPLC-DAD-TOF/MS as a system for accurate and rapid detection of anti- Aβ fibrillation inhibitors from TCMs.

## Discussion

Accumulation of Aβ in senile plaques and hyperphosphorylated tau in NFTs are the two main pathological characteristics in the brain of AD patients. Experimental evidence has suggested that Aβ is an important biomarker for diagnosis and drug target of AD, based on the amyloid hypothesis of AD (Thakur et al., 2009; Selkoe and Hardy, 2016; Tamagno et al., 2017). The Aβ (1–42) peptide derived from APP through the cleavage of γ-secretases and β-secretases, is the predominant aggregated and neurotoxic form of Aβ found in the brain of AD patients due to its two additional hydrophobic amino acids (Butterfield et al., 2013; Xiao et al., 2015). The excessive formation and failure on the clearance of Aβ, can lead to the formation of self-aggregated Aβ in different forms such as oligomers and neuritic plaques, which possess neurotoxicity and can affect axon function via oxidative stress (Lecanu et al., 2006), altered electrochemical signaling (Walsh et al., 2002) or *N*-methyl-D-aspartate (NMDA) receptor-mediated excitotoxicity (You et al., 2012), which can lead to impaired cognitive and memory functions (Palop and Mucke, 2010). Current pharmacological interventions to AD include the use of cholinesterase inhibitors, NMDA receptor antagonists and neurotrophic factors (Masters et al., 2015). In addition, inhibitors targeting the different structures of Aβ have also been widely studied (Kepp, 2012; Doig and Derreumaux, 2015). For example, carnosine (β-alanyl-L-histidine) can interact with Aβ (1–42) monomer and inhibit Aβ aggregation (Attanasio et al., 2013). As an inhibitor of Aβ oligomers, both curcumin and resveratrol can bind to the N-terminus (residues 5–20) of Aβ 42 monomers (Fu et al., 2014), while BAN2401 (humanized version of mAb158) decreases the level of soluble Aβ protofibrils (Lannfelt et al., 2014). A recent report has suggested that the human innate immune peptide LL-37 can bind to Aβ and modulate the formation of the Aβ fibril (De Lorenzi et al., 2017). However, current available drugs or treatments are only symptomatic which mainly target the modulation of cognitive ability or neuropsychiatric symptoms.

As amyloid deposition starts early, before the onset of dementia symptoms (Lannfelt et al., 2014), preventive drugs are particularly important in AD treatment. With the advantages of multi-targets and high drug safety, TCMs are commonly used as a preventive prescription for aged-related degenerative diseases, therefore, identifying potential active compounds from TCMs has become an important strategy for AD therapy (Pan et al., 2013). TCMs have been used for the treatment of AD for a long time in China. For example, huperzine A, a lycopodium alkaloid isolated from *huperzia serrata*, could improve AD symptoms through inhibiting acetylcholinesterase (AChE) activity (Zhu et al., 2010). The leaf extract of Ginkgo biloba (EGb 761) could protect hippocampal neurons against cytotoxicity induced by Aβ fragments (Bastianetto et al., 2000; Wu et al., 2011). However, potential drug discoveries from TCMs still encounters challenges in the quick and precise identification of bioactive components from its complex herbal formulation (Liu et al., 2017).

SB has been reported for its protective roles in the improvement of learning ability (Heo et al., 2009) and brain injury (Hwang et al., 2011; Lin et al., 2011) possibly via its anti-oxidation, -inflammation (Yune et al., 2009) and -apoptosis properties (Jeong et al., 2011). Although a previous study has reported only the moderate neuroprotectivity of SB extract for Aβ insult (Kim et al., 2007), possibly due to the low testing concentration, other studies have confirmed that flavonoids from SB play a protective role in the recovery of neurological functions and in the prevention of AD (Gasiorowski et al., 2011; Gao et al., 2013; Vauzour, 2014). For example, total flavonoids from SB can protect neuronal damage and improve memory deficits induced by cerebral ischemia in rats (Shang et al., 2006; Cao et al., 2016). Additionally, a flavonoid composition (UP326) containing baicalin can help to maintain memorizing and processing abilities of aged animals (Yimam et al., 2016). As the major type of flavonoids in SB, baicalein and baicalin ameliorated cognitive impairment in AD animal models (Wei et al., 2014; Chen et al., 2015). In fact, the recent development of plant-derived drugs has received considerable attention in the treatment of AD (Kumar and Nisha, 2014), therefore, based on the previous literature on SB, the development of accurate detection methods of active components from herbal plants is performed in this study by choosing baicalein and baicalin as the study subjects.

It is noteworthy that traditional bioactivity guided purification of single active compounds from TCMs is time-consuming, laborious, costly and has low sensitivity. Although ThT fluorescence dye is a commonly used fluorescent probes for Aβ fibril detection, it possesses the disadvantages of low specificity and sensitivity (Roberti et al., 2009; Kitts et al., 2011; Li et al., 2013). Cell-based detection methods have been developed by expressing the GFP-Aβ 42 fusion protein in E. coli, which allows the rapid high throughput screening of inhibitors on Aβ aggregation (Kim et al., 2006). Furthermore, the advances in the use of affinity chromatography in conjunction with highly sensitive detection technology has also facilitated the detection of bioactive compounds from TCMs. For example, biolayer interferometry accurately measures the real-time biomolecular interactions in the cells-free system, which has been used to identify D-enantiomeric peptide D3 and its derivatives as Aβ oligomers inhibitors (Concepcion et al., 2009; Klein et al., 2016). Nuclear magnetic resonance spectroscopy (NMR), was used to identify inhibitors on Aβ fibrillation from a compound library with high sensitivity and speed (Sievers et al., 2011). Furthermore, mass spectroscopy coupled with liquid chromatography (LC) was used for rapid detection of small molecules such as epigallocatechin gallate (EGCG), hemin and tramiprosate that could bind to Aβ precursors (Young et al., 2015). Although some of the current detection methods show high sensitivity and analytical speed, these methods work best only in targeting single compounds, but not to extract of TCMs that contain multiple chemical components.

UHPLC-DAD-TOF-MS is a common analytical equipment that provides accurate mass analysis including profiling, characterization, identification and quantification of a mixture of chemical molecules, based on their molecular weights, with highly sensitive. Therefore, it serves as a useful tool for the identification of single components from complicated chemical compositions like the extract of TCMs (Hossain et al., 2010). We have previously reported the successful identification of the natural methylglyoxal (MGO) scavenger from *Polygonum cuspidatum* and the autophagy inducer from *Radix polygalae* using UHPLC-DAD-MS and UHPLC-DAD-TOF-MS, respectively (Tang et al., 2013; Wu et al., 2013). According to the published reports, the peak area of the bioactive inhibitors including small molecule and peptide in the NMR spectrum decreased significantly upon their binding to the Aβ or tau fiber (Sievers et al., 2011; Jiang et al., 2013). Based on the above observation, we have therefore hypothesized and confirmed that the components in TCMs, with an inhibition effect on Aβ fibrillation, must possess binding affinity to the Aβ fibril, and these bioactive components can be successfully detected using UHPLC-DAD-TOF-MS via chromatogram analysis. In the present study, although the method was successfully applied on SB-TEE and its major known chemical components, further verification on different TCMs along with a biological evaluation is required to further validate accuracy. In additional to MS, which provides information on the possible potency and accurate identifies of the herbal chemicals, combinational use of column chromatography and NMR technology, for the analytical isolation and identification of novel or low abundance active compounds presented in SB or other herbs, are required.

## Conclusion

**FIGURE 8:**
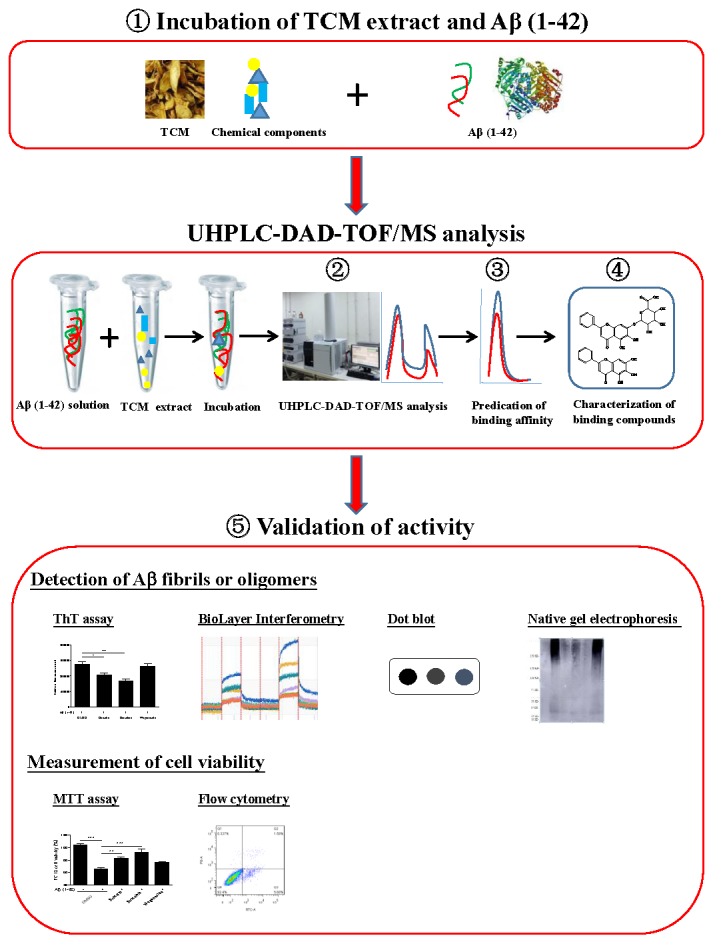
Schematic diagram for the rationale, design and characterization of Aβ fibril inhibitors from herbal medicines. 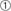 The incubation of Aβ (1–42) solution with TCMs extract. 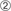 Analysis of the pre-incubated Aβ (1–42)-TCM extract mixture using Agilent 6230 UHPLC-DAD-TOF/MS instrument. 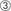 Chemical analytical prediction on the binding propensity of the compounds by UHPLC-DAD-TOF/MS. 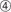 Identification of the Aβ (1–42) binding chemical components from TCMs with their accurate mass identified and confirmed from literatures. 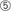 Bioassay-validation of the Aβ (1–42) peptide-binding propensity of the identified compounds.

In conclusion, up to now, there are still no effective and reliable detection methods for the inhibitors of β-amyloid fibrillation from complicated chemical composition such as TCMs. As shown in Figure 8, based on the fact that the peak area of the compounds on the chromatogram are reduced upon their binding with Aβ fibril, we hypothesized that the components in TCMs, that have binding propensity for Aβ, can be identified using UHPLC-DAD-TOF/MS. Through the effective and accurate isolation of the active compounds from TCMs, UHPLC-DAD-TOF-MS has therefore proved to be a useful method for identifying the bioactive compounds in TCMs with anti-Aβ fibrillation effects. In the near future, the method proposed in this study may also be applied for the precise screening of inhibitors on other AD-related proteins such as tau, which may facilitate AD drug discovery from TCMs.

## Author Contributions

LY and A-GW conducted the experiments and drafted the manuscript. A-GW and WZ conducted the chemical analysis experiments. LY, L-QQ, BT, NZ, and H-MW conducted the bioassay experiments. V-WW and D-LQ conceived and designed the experiments. BL revised the whole manuscript. QW and BL conceived the idea and designed the experimental plan. All authors reviewed the above manuscript.

## Conflict of Interest Statement

The authors declare that the research was conducted in the absence of any commercial or financial relationships that could be construed as a potential conflict of interest.
