# Supplementary material for: The New Application of UHPLC-DAD-TOF/MS in Identification of Inhibitors on β-Amyloid Fibrillation From Scutellaria baicalensis
Source: Front Pharmacol. 2019 Mar 18;10:194. doi: 10.3389/fphar.2019.00194 (PMC6431657; doi:10.3389/fphar.2019.00194)
Supplement: Supplementary file 1 [file Image_1.pdf]

The new application of UHPLC-DAD-TOF/MS in identification of inhibitors on β-amyloid fibrillation from Scutellaria baicalensis

Lu Yu[1,2,5†], An-Guo Wu[1,3†], Vincent Kam-Wai Wong[1], Bin Tang[1], Hui-Miao Wang[1], Qiong Wang[4,5,6*], Betty Yuen-Kwan Law[1*]

[1] State Key Laboratory of Quality Research in Chinese Medicine, Macau University of Science and Technology, Macau, China

[2] Laboratory of Medical Chemistry, Department of Chemistry, School of Basic Medical Sciences, Southwest Medical University, Luzhou, Sichuan Province, China

[3] Laboratory of Chinese Materia Medica, Department of Pharmacology, School of Pharmacy, Southwest Medical University, Luzhou, Sichuan, China

[4] Affiliated Traditional Chinese Medicine Hospital, Southwest Medical University, Luzhou, Sichuan Province, China

[5] Sino-Portugal Traditional Chinese Medicine International Cooperation Center, Southwest Medical University, Luzhou, Sichuan Province, China

[6] School of Pharmacy, Southwest Medical University, Luzhou, Sichuan Province, China

† These authors contributed equally to this work.

***Corresponding authors:**

Dr. Betty Yuen-Kwan Law

E-mail: yklaw@must.edu.mo.

Prof. Wang Qiong

E-mail: wqimplad@126.com

1 Supplementary Figure

Figure 5 Inhibitory effect of SB-TEE and selected compounds on Aβ (1-42) fibrillation by dot blot assay and native gel electrophoresis analysis. The full-length images of dot blot and native gel electrophoresis as shown in **Figure S1.**

Figure S1

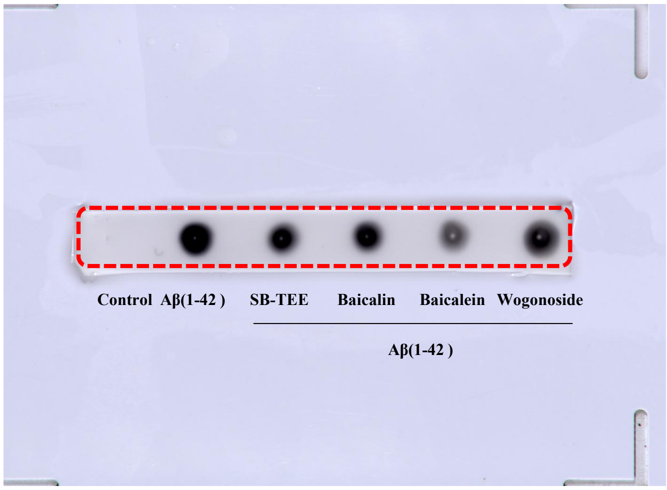
Control
Aβ(1-42)
SB-TEE
Baicalin
Baicalein
Wogonoside
Aβ(1-42)

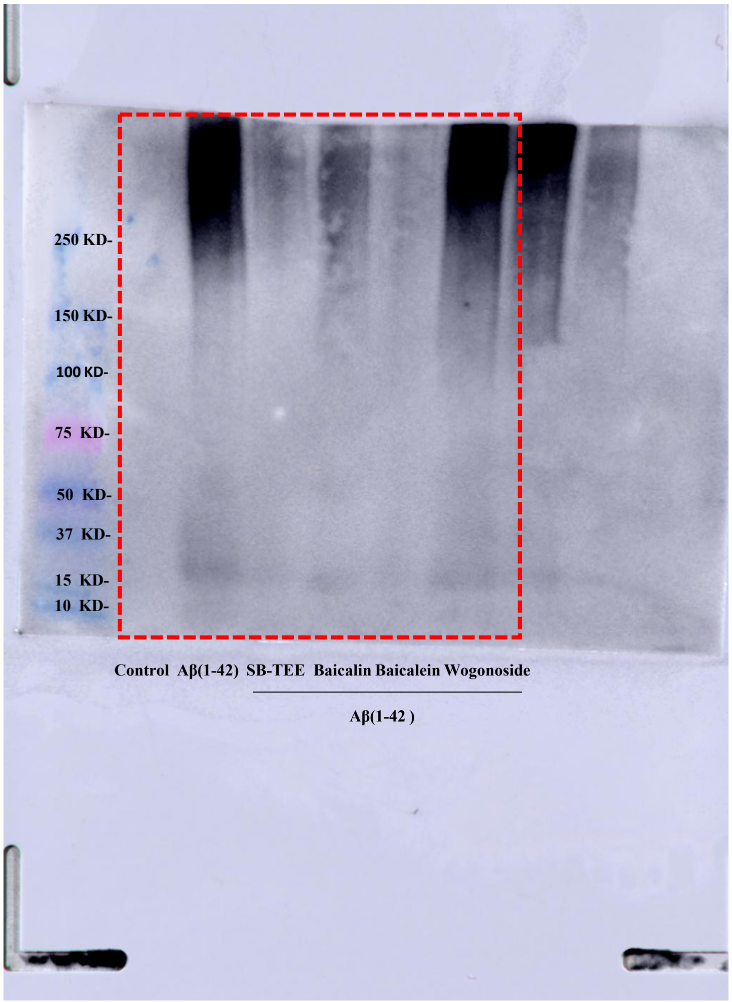
250 KD-
150 KD-
100 KD-
75 KD-
50 KD-
37 KD-
15 KD-
10 KD-
Control
Aβ(1-42)
SB-TEE
Baicalin
Baicalein
Wogonoside
Aβ(1-42)
